# Parental phonological memory contributes to prediction of outcome of late talkers from 20 months to 4 years: a longitudinal study of precursors of specific language impairment

**DOI:** 10.1186/1866-1955-4-3

**Published:** 2012-02-08

**Authors:** Dorothy VM Bishop, Georgina Holt, Elizabeth Line, David McDonald, Sarah McDonald, Helen Watt

**Affiliations:** 1 Department of Experimental Psychology, University of Oxford, South Parks Road, Oxford, OX1 3UD, UK

## Abstract

**Background:**

Many children who are late talkers go on to develop normal language, but others go on to have longer-term language difficulties. In this study, we considered which factors were predictive of persistent problems in late talkers.

**Methods:**

Parental report of expressive vocabulary at 18 months of age was used to select 26 late talkers and 70 average talkers, who were assessed for language and cognitive ability at 20 months of age. Follow-up at 4 years of age was carried out for 24 late and 58 average talkers. A psychometric test battery was used to categorize children in terms of language status (unimpaired or impaired) and nonverbal ability (normal range or more than 1 SD below average). The vocabulary and non-word repetition skills of the accompanying parent were also assessed.

**Results:**

Among the late talkers, seven (29%) met our criteria for specific language impairment (SLI) at 4 years of age, and a further two (8%) had low nonverbal ability. In the group of average talkers, eight (14%) met the criteria for SLI at 4 years, and five other children (8%) had low nonverbal ability. Family history of language problems was slightly better than late-talker status as a predictor of SLI.. The best predictors of SLI at 20 months of age were score on the receptive language scale of the Mullen Scales of Early Learning and the parent's performance on a non-word repetition task. Maternal education was not a significant predictor of outcome.

**Conclusions:**

In this study, around three-quarters of late talkers did not have any language difficulties at 4 years of age, provided there was no family history of language impairment. A family history of language-literacy problems was found to be a significant predictor for persisting problems. Nevertheless, there are children with SLI for whom prediction is difficult because they did not have early language delay.

## Background

**Figure 1:**
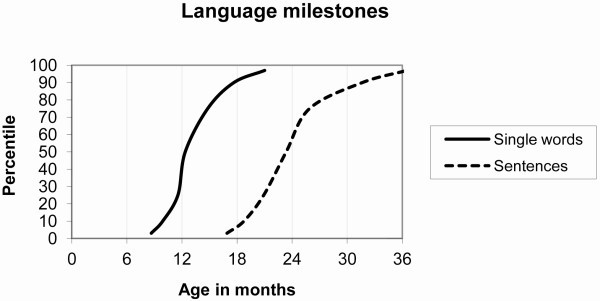
**Normative data on language milestones from Neligan and Prudham **[2]. Note that for Words, the interval between the 50th and 95th percentile is considerably greater than that between 5th and 50th percentile.

The ease and speed with which children master their native language has been widely commented on, most memorably by Pinker ([1]. p. 29), who noted: 'In general, language acquisition is a stubbornly robust process; from what we can tell there is virtually no way to prevent it happening short of raising a child in a barrel'. Nevertheless, there is a fairly wide spread of ages at which mastery of first words and sentences occurs (Figure 1), with children in the 10th centile (that is, top end) of language development talking in sentences at 18 months of age, and those in the 90th centile producing at most a handful of single words at this age [2]. The study of Neligan and Prudham, although old, is valuable because it was based on all children born in a large English city (Newcastle upon Tyne) over a 2-year period. Figure 1 also illustrates the skew in the distribution of age at first words: the difference between the 3rd and 50th centiles is around 4 months, whereas the difference between the 50th and 90th centiles is twice as large.

We know that in some children, a late start in language acquisition is a harbinger of long-term problems. Delay in language milestones is a common reason for parents to consult a physician, and can be the first indication of a serious problem such as severe hearing loss, nonsyndromic intellectual retardation or autistic disorder [3,4]. Furthermore, children who are subsequently diagnosed with specific language impairment (SLI) in the absence of other developmental difficulties were usually late talkers. For instance, in a study of children at a residential school for those with specific speech and language impairments, Haynes and Naidoo [5] found that only 12% had produced their first words by 17 months of age. Parents of children with SLI often complain that their early concerns were not taken seriously, and that they are told that the child would 'grow out of it'. This view has been articulated in a UK government report into provision for children with speech and language difficulties [6].

On the other hand, there is evidence that many late talkers do 'grow out of it', and catch up with their peers after a slow start, provided their language delay is not associated with other developmental difficulties. This conclusion is indicated by a simple consideration of base rates, that is, the relative frequency of late talkers and SLI in the population. We can see from the norms in the study of Neligan *et al*. [2] that around 10% of children have not produced their first words by 18 months of age. Severe SLI, of the kind studied by Haynes and Naidoo [5], probably affects around 3% of children at most, and the data from this study indicate that 88% of these children had not produced first words by 18 months, which we will take as our definition of 'late talker'. This means that in a population of 1000 children, we will expect there to be 100 late talkers and 30 children with severe SLI. Of the 30 children with severe SLI, 88%, that is, 26 children, will be late talkers, but this means that three out of four late talkers will not have severe SLI. This example could be criticized because the predictions depend so crucially on the estimated prevalence of SLI, and that in turn depends on severity. However, there is also empirical evidence to support the idea that most late talkers do not have adverse outcomes.

'Late bloomers'', that is, children who make good progress in language after a slow start, are well-documented in the literature. For instance, one study followed 26 children aged 2 years old, who were recruited because their parents reported that they understood complete sentences but could say only a few words [7]. Five months after the initial assessment, around one-third of the children still had problems, one-third had made some improvement, and one-third were in the normal range. Another study followed 10 children who scored in the bottom 10% for expressive vocabulary at the ages of 18 to 29 months [8]. One year after initial assessment, six had 'caught up', but the remaining four still had delayed language. Similar figures were reported by Rescorla and Schwartz [9], who followed up 25 boys who had specific expressive language delay when first seen at 24 to 31 months of age. By follow-up at 3 to 4 years old, ten boys no longer had impaired language and two of these were above average in their utterance length. However, the remaining 15 children still had significant language delays. Although the proportion of late talkers who have clinically significant language impairment appears to decline with age [10], some children fail to catch up, and still have persisting problems well into middle childhood: for instance, Moyle *et al*. [11] reported that 37% of late talkers were receiving speech and language therapy (SALT) at 5 years of age, and Rice *et al*. [12] found that around 20% of late talkers had language impairment at 7 years of age. Measures of morphosyntax appear to be particularly sensitive in revealing persisting language deficits in late talkers [12,13].

Such findings pose a quandary for those who carry out intervention with language-impaired children. In general, it is recognized that early intervention is desirable, insofar as it may help avert secondary social, emotional, or behavioral difficulties that can develop in response to communication failure. There is also a biological argument in favor of early intervention, namely that it is easier to influence the trajectory of development while the brain is still plastic. An analogy may be made with vision; it is well-recognized that amblyopia ('lazy' eye) needs to be corrected early in life, otherwise neural pathways subserving vision do not develop normally, and the potential for good vision in the 'lazy' eye is lost [14]. In the domain of language, there are no animal models, but evidence for a sensitive period comes from second-language learning, where native-level proficiency becomes harder to achieve with age [15]. In addition, Lenneberg [16] argued that ability to develop normal language after a left-hemisphere brain injury declined throughout childhood. Both sets of findings suggest that it may be difficult to learn language once patterns of brain connectivity have become entrenched.

Despite the evident advantages of early intervention, matters are complicated by the phenomenon of 'late bloomers'. On the one hand, it is undesirable to offer therapy to a child who will make good progress in their own time, especially if intervention resources are scarce and costly. Furthermore, more harm than good may result if a parent is made anxious, or the child is made to feel self-conscious or abnormal, when their development is likely to move within the normal range spontaneously. What is needed, therefore, is a way to distinguish between late talkers who are just late bloomers, and those who are at high risk of long-term problems.

Dale *et al*. [17] found that although parental report of language skills at 2 years of age was significantly related to language outcome at 3 or 4 years, classification of outcome for individuals was far too inaccurate to be clinically useful. Furthermore, those whose language difficulties persisted were not necessarily those with the poorest scores at 2 years. O'Hare [18] noted the recommendation of the UK National Screening Committee Child Health Subgroup on speech and language delay, which stated that, because isolated expressive language delay presenting before the age of 3 years has a good prognosis, an approach of watchful waiting was appropriate unless the parent was very worried. More recently, Paul and Roth [19] reviewed the literature on this question, concluding that 75% of children identified as late talkers at 18 months of age will move into the normal range on standardized language measures by 3 years of age. They proposed a number of 'red flags' that clinicians should note as predictive of poor outcome in late talkers, including presence of delays in comprehension and expression, and a family history of language delays or reading problems. This does not necessarily mean that catch-up is complete in other children. As shown in series of follow-ups, late-talking middle-class children with purely expressive delays at 2 years may continue to do poorly relative to high-performing SES-matched controls, but by school age and beyond most of these children will obtain language and literacy scores well within normal limits [20].

Since Paul and Roth's review, two large-scale epidemiological studies have been published on this topic. These studies, like the earlier population studies of Rice *et al*. [12] and Westerlund *et al*. [21], have confirmed the very varied outcomes of late talkers, with most of the children identified as late talkers at 2 years moving into the normal range over the next year or two. Although significant predictors were found, the accuracy of prediction of outcome in individual cases was fairly weak. In the Generation R Study [22], Henrichs *et al*. used a short Dutch version of the MacArthur Communicative Development Inventory (CDI), and found that, although there were highly significant correlations between expressive vocabulary at 18 months and 30 months, the sensitivity and specificity of late-talker status was very poor at predicting language status at 30 months, and inclusion of parental, perinatal, and demographic variables did not substantially improve prediction. The poor prediction was not only due to the numbers of late talkers with good outcome; the sample also included notable numbers of children who appeared to have language impairment at 30 months of age, but who had not shown signs of language delay at 18 months. In the second of these studies, the Early Language in Victoria Study (ELVS), parental report of language development was obtained at 2 years of age, with follow-up assessments of language and nonverbal skill at 4 years of age, for 1596 children in Melbourne, Australia [23]. The data at 4 years of age were used to identify children with SLI, using the criteria of a score more than 1.25 SD below the mean on the Clinical Evaluation of Language Fundamentals - Preschool 2 and of nonverbal ability within 1.25 SD of the mean. Children with English as a second language, autism, or hearing loss were excluded. Predictors of SLI status were first examined without considering language status at 24 months; significant predictors were male gender, maternal education, socioeconomic status, family history of speech and language problems, and maternal vocabulary. Prediction was improved when late-talking status at 2 years was added to the regression model, but it was not stated what proportion of late talkers met the criteria for SLI. It is noteworthy that this study found that a family history of language or literacy impairment was a significant predictor of outcome, consistent with smaller-scale studies investigating outcomes of children with a family history of dyslexia [24] or SLI [25].

In the current study, we performed an in-depth assessment of a sample of 20-month-old children, enriched with late talkers, who were then given an assessment at 4 years of age to identify those meeting the criteria for SLI. Our goal was to see whether it would be possible to predict which late talkers would have good language outcomes, and whether including assessments of parental language skills would improve the predictive power.

## Methods

### Ethics approval

This project was approved by Oxfordshire Research Ethics Committee A (file number A03.025). Parents gave signed consent for their own and their child's participation.

### Participants

Children were recruited from a database of volunteer families contacted via a local maternity ward and through local toddler groups. Children with serious birth complications were not included. Mothers whose children were 18 to 19 months of age were sent the Oxford University Communicative Development Inventory (OCDI; a British adaptation of the MacArthur-Bates Communicative Development Inventory [26]) and invited to volunteer for the study. The OCDI was supplemented with the question 'Has anyone related to your child (e.g. brother, sister, parent) had difficulties with language or reading?'. From the families responding, we selected 26 children who were late talkers, defined as having an expressive OCDI of more than 1 SD below the mean (≤ 10 words), and 70 average talkers, scoring between the 20th and 75th centile (range 13 to 196 words). Children whose parents reported a positive family history for language or reading impairment in a first-degree relative (parent or sibling of the child) were given preference during the selection of children for inclusion in the study, and comprised 21 (30%) of the average talkers and 7 (27%) of the late talkers. All children were reported to have normal hearing and vision, and no more than occasional exposure to a language other than English. These children were seen for a language and cognitive assessment at around 20 months of age. Parents were approached again just before the child's fourth birthday, and invited to participate in a follow-up stage of the study at which their child's language abilities would be assessed. In total, 24 of the original late talkers and 59 of the average talkers were available for follow-up. One of the average talkers was excluded at this stage because a sensorineural hearing loss had been discovered, leaving a sample of 58 in this group.

### Assessments at 18 to 20 months of age

#### The Oxford University Communicative Development Inventory

The OCDI was used to select late talkers for the study. It is a standardized parental report of a child's ability to comprehend and produce lexical items from a list of 437 words, organized into 21 categories. Normative data from 355 children aged 18 to 24 months [26] were used to convert OCDI data for vocabulary production and comprehension into age-adjusted *z*-scores (standardized residuals), using the linear regression of the OCDI raw score on age. The OCDI was completed 4 to 6 weeks before the family visited the laboratory for more detailed assessment.

#### The Vineland Adaptive Behavior Scales

We used the second edition of the Vineland Adaptive Behavior Scales (VABS), [27], which is a standardized parental interview used to evaluate the child's development in the areas of communication, socialization, daily living, and motor skills.

#### The Mullen Scales of Early Learning

The Mullen Scales of Early Learning (MSEL) [28] is a standardized child-based assessment that provides scores for motor skills, visual reception, expressive language, and receptive language.

### Assessments at 4 years of age

#### Wechsler Preschool and Primary Scales of Intelligence

The two subtests of block design and matrices from the Wechsler Preschool and Primary Scales of Intelligence, third UK edition (WPPSI-III) [29] were used to assess nonverbal ability, Results from these were combined to give a pro-rated estimate of performance IQ (PIQ).

#### British Ability Scales

Two subtests were also given from the British Ability Scales (BAS) [30]: verbal comprehension and naming vocabulary. In the verbal comprehension test, the child is asked to point to named items, manipulate objects according to instructions, or select a picture to match a spoken utterance. In the naming vocabulary test, the child is asked to name pictured objects. Scores were transformed to *z*-scores using published norms.

#### The Grammar and Phonology Screening test

The Grammar and Phonology Screening (GAPS) Test [31] assesses sentence repetition and non-word repetition. This test was used as specified in the manual except that the child was asked to repeat items to a furry toy squirrel rather than to a cardboard cutout alien, which had frightened some children in pilot testing. Scores were transformed to *z*-scores by transforming published percentiles, treating the maximum score as equivalent to *z *= 2.0.

#### The Bus Story Test

The Bus Story Test [32] is a narrative task in which the child is told a short story accompanied by pictures, and then asked to retell it while reviewing the pictures. It generates two scores: an information score, which represents how much of the story content is given, and a sentence length score, which is the mean length of the five longest complete sentences. The UK version of the materials was used, but because the UK manual reports only normative mean and SD for the information score, the closely similar US version was used to derive age-scaled scores for sentence length.

#### Test of Early Grammatical Impairment

Two probes (third person singular and past tense) from the Test of Early Grammatical Impairment (TEGI) [33], were used to elicit verb inflectional endings from the child. This test is scored in an all-or-nothing fashion, according to whether the proportion of inflected items is in line with age expectation or not.

#### Literacy skills

Early literacy skills were assessed by asking children to name 13 single letters (b, c, d, g, h, i, l, k, l, o, t, x, y *z*) written on cards. They were credited with one point if they could produce either the name or the sound the letter made. In addition, they were given the Early Word Reading Test, an experimental word list [34] consisting of 50 words found in the sight-word vocabulary of early readers. The words were arranged in order of frequency, and testing stopped when the child was unable to name four of five consecutive items.

#### Communication skills

Everyday communication skills were assessed using the Children's Communication Checklist (CCC)-2 [35]. This was completed by a parent, usually the mother. This checklist is designed to measure aspects of communication and related skills that are not easy to assess using direct examination. It has been standardized on 542 British children aged 4 to 16 years, and yields a General Communication Composite (GCC) that has been shown to be effective in distinguishing children with clinically significant communication difficulties from those who are developing typically children [36]. For the current study, the GCC was transformed to a *z*-score on the basis of published norms.

### Assessment of parental characteristics

The parent who accompanied the child (in all but three cases, the mother) was invited to complete the non-word repetition subtest of the NEPSY (A Developmental NEuroPSYchological Assessment [37]) at both the 20-month and 4-year assessments. This subtest consists of 13 non-words, ranging in length from two to five syllables, all but one of which contains consonant clusters. The participant hears each recorded non-word over headphones and repeats it immediately, with accuracy being scored in terms of number of syllables correct, out of 46 in total. All items are given to all participants. The test is standardized only up to 12 years of age on a US sample, so our own data, from typical adults using this version with items recorded by a British English speaker [38], were used to convert scores to standard scores. According to the NEPSY manual [37], this subtest has reasonable test-retest reliability, with a correlation of 0.67 for the oldest normative group (12 to 13 years) over an interval of 2 to 10 weeks. Because the follow-up assessment was performed in the home setting, we made a procedural change to minimize the effect of distractions. Instead of using the standard method, where the adult listens over headphones to a continuous sound file with non-words spoken at 5-second intervals, the experimenter controlled the timing of presentation of the sound files. This made it easier to ensure the participant was focused on the task. As discussed below, this apparently minor change to the administration appeared to have a large effect on performance. During the assessment for 4-year-olds, the parent was also asked to complete sets A and B of the multiple choice version of the Mill-Hill vocabulary scale [39], a written test of word meanings. In addition, the parent completed a short questionnaire about the child's early medical history, concerns about speech and language development, and educational placements. We also recorded the age at which the mother left full-time education. The parent provided information about whether any other relatives had problems with speech, language, or reading, and a family history was recorded if there was a first- degree relative who was reported to have dyslexia or articulation problems, was late in talking, or had received SALT for a condition other than lisp or stutter. Inclusion of information about literacy and language problems in relatives is justified by research indicating a high level of language deficits in children of dyslexic parents [40,41]. Our definition of positive family history was closely similar to that used in the ELVS [42].

### Classification of children's language status at 4 years

The test battery for 4-year-olds yielded nine language measures: BAS verbal comprehension, BAS naming, GAPS sentence repetition, GAPS non-word repetition, Bus Story information, Bus Story sentence length, TEGI third person singular, TEGI past tense, and GCC from the CCC-2. Although the test scores for 4-year-olds are continuous, a categorical outcome measure is more useful for evaluating accuracy of prediction in a clinical context [43].

There is no 'gold standard' definition for SLI, and varied psychometric criteria have been adopted in the past. One approach is to take an average language score and set a cutoff for impairment, but this has the disadvantage that it would miss a child who had a severe but selective impairment, for example, in grammatical morphology. Because previous research had suggested that late talkers may show uneven profiles of language outcome [44], we adopted a criterion that would identify children with selective deficits in particular aspects of language, requiring that for a child to be identified with SLI, they had to be impaired on two or more language measures [38]. In the past, we have found that this definition yields good agreement with external evidence of clinical or parental concern [38]. For the TEGI measures, the test manual does not give percentiles, but categorizes scores in a binary fashion, so on this test, impairment was defined as a score categorized as below age expectation. For the other measures, a quantitative cutoff of impairment corresponding to a score 1 SD below the mean (16th percentile) was used. This is less stringent than the 10th centile used by Barry *et al*. [38], but was adopted to achieve adequate numbers of SLI cases for analysis. We thus had nine binary-coded (impaired/unimpaired) language measures for each child that were used to classify them into four groups, as follows:

• SLI: impaired on at least two language measures, with WPPSI PIQ of 85 or above (n = 15; 12 of whom also met the more stringent 10th centile cutoff).

• Nonspecific language impairment (NLI): impaired on at least two language measures, with WPPSI PIQ of 84 or below (n = 4).

• Low nonverbal ability (LNV): WPPSI PIQ of 84 or below, but no more than one impaired language measure (n = 3).

• Typical development (TD): WPPSI PIQ of 85 or above, and no more than one impaired language measure (n = 60).

Because there were few children with low nonverbal ability (NLI and LNV), they were excluded from all subsequent analyses, although their descriptive data are shown for completeness.

## Results and discussion

### Characteristics of the sample at 4 years

Various parameters were assessed for the samples, including numbers of boys and girls in each category at 4 years of age, and data on whether the child had received SALT or the parent had concerns about speech or language development at this age (Table 1). The gender ratio did not differ significantly between the TD and SLI groups, χ^2 ^= 0.87, degrees of freedom (d.f.) = 1, *P *= 0.35. As might be expected, there was a highly significant difference between these two groups in the rate of parental concern about language or SALT involvement, χ^2 ^= 9.61, d.f. = 1, *P *= 0.002, providing some validation for the categorization. Nevertheless, for just over half those meeting the criteria for SLI, there was no indication of parental concern or SALT.

**Table 1:** Characteristics of the sample at 4 years, divided according to outcome.

	TD	SLI	NLI	LNV	**Cohen *d***, TD vs SLI
Children, n	60	15	4	3	-
Male, %	0.53	0.67	0.50	0.33	-
SALT^a^/language concern, %	0.08	0.40	0.50	0.00	-

Test scores,^b ^mean ± SD					

WPPSI, block design	0.78 ± 0.97	0.38 ± 0.84	-1.92 ± 0.83	-0.11 ± 0.51	0.43
WPPSI, matrices	0.29 ± 0.72	-0.29 ± 0.68	-1.00 ± 0.27	-1.56 ± 0.51	0.83
BAS, verbal comprehension	0.53 ± 0.87	-0.30 ± 0.85	-0.79 ± 0.42	-0.23 ± 1.00	0.97
BAS, naming vocabulary	1.31 ± 0.75	0.27 ± 1.07	-0.93 ± 0.96	0.54 ± 0.91	1.28
GAPS, sentence repetition	0.97 ± 0.93	-0.16 ± 0.87	-0.87 ± 0.29	0.43 ± 0.78	1.25
GAPS, non-word repetition	0.70 ± 0.82	-0.59 ± 1.27	-0.79 ± 0.91	-0.26 ± 1.08	1.42
Bus story, information	0.17 ± 1.19	-1.48 ± 0.71	-1.78 ± 0.48	-1.30 ± 0.52	1.50
Bus story, sentence length	0.73 ± 1.22	-0.68 ± 0.82	-1.30 ± 0.76	-0.83 ± 0.61	1.24
CCC-2, general communication	0.13 ± 0.83	-0.83 ± 0.61	-0.57 ± 0.46	-0.72 ± 0.74	1.23
TEGI, third singular	0.93 ± 0.14	0.62 ± 0.36	0.33 ± 0.38	0.81 ± 0.19	1.56
TEGI, past tense	0.94 ± 0.09	0.72 ± 0.29	0.57 ± 0.25	0.86 ± 0.12	1.48

^a^Abbreviations: BAS, British Ability Scales; CCC-2, Children's Communication Checklist, version 2; GAPS, Grammar and Phonology Screening Test; GCC, General Communication Composite; LNV, low nonverbal; NLI, nonspecific language impairment; SALT, speech and language therapy; SLI, specific language impairment; TD, typical development; TEGI, Test of Early Grammatical Impairment; WPPSI: Wechsler Preschool and Primary Scale of Intelligence.

^b^Scores shown as *z*-scores based on test norms, except for TEGI, where scores are proportion correct.

The mean scores on the tests used to define the outcome groups were also analyzed (Table 1). Because the tests were used to categorize the children, group means were expected to differ, but it is nevertheless of interest to consider which measures were most effective in distinguishing groups. The two groups with low nonverbal ability contained too few children to include in analysis, but their means are included for completeness. Effect sizes for the TD/SLI contrast (Table 1) were substantial for all the language measures. These were computed using the standard deviation for pooled samples. Note, however, that for some tests the TD *z*-score mean was well above zero, and for the BAS in particular, this could reflect outdated norms. In addition, several of the language test means for the SLI group were only slightly below average; this is explicable in terms of the method of identifying SLI, which requires the child to be impaired on at least two measures, so not all children with SLI were impaired on all measures. On average, a child in the TD group had a score (mean ± SD) in the impaired range (see above) of 0.35 ± 0.48) of the 9 measures, whereas a child in the SLI group scored in the impaired range for 3.1 ± 1.67 measures.

At this age, children's literacy development was only at the earliest stages, but there was nevertheless a striking difference between the TD and SLI group in terms of the number of letters recognized, with mean ± SD for TD being 7.6 ± 4.22 and for SLI being 3.5 ± 3.60 (*t *= 3.5, d.f. = 73, *P *< 0.001). Only six children, five of whom came from the TD group, could read any of the words from the Early Word Reading Test.

### Outcome at 4 years in relation to late-talker status and family history

We assessed the relationship between SLI at 4 years of age and late-talker status at 18 to 20 months, subdivided according to whether there was a positive family history (FH+) of language/literacy problems (Table 2). The children with low nonverbal ability (NLI and LNV) are shown as a single group for completeness, but were excluded from subsequent analyses. The overall χ^2 ^test on data showed a non-significant trend for association between classifications at the two ages (χ^2 ^= 7.23, d.f. = 3, *P *= 0.065) (Table 2). However, on inspection the association with SLI outcome appeared stronger for FH+ than for late-talker statusThis was confirmed by subsidiary analyses. If late-talker status was dropped from the analysis, to focus only on the association between FH+ and SLI outcome, this gave a significant association (χ^2 ^= 4.53, d.f. = 1, *P *= 0.033). By contrast, when children were categorized by late-talker status, ignoring FH+, the trend for an association with outcome fell short of significance (χ^2 ^= 2.72, d.f. = 1, *P *= 0.10).

**Table 2:** Frequencies: language status at 4 years vs. 18 to 20 months, and family history

Language status at 18-20 months	Language status at 4 years, n (%)			Total, n
		
	TD	SLI	LNV	
AT, no FH^a^	34 (91.9)	3 (8.1)	2 (5.1)	39
AT + FH	11 (68.8)	5 (31.3)	3 (15.8)	19
LT, no FH	11 (73.3)	4 (26.7)	2 (11.8)	17
LT + FH	4 (57.1)	3 (42.9)	0 (0)	7
Total	60	15	7	76

^a^Abbreviations: AT, average talker; FH, family history; LNV, low nonverbal; LT, late talker; SLI, specific language impairment; TD, typical development.

We next considered how the initial assessments related to outcome at 4 years, calculating the means of variables measured at 18 to 20 months, together with the effect size of the difference between those with TD and SLI outcomes at 4 years of age (Table 3). There was a pattern for all mean scores to be higher for the TD than the SLI group, but most effect sizes were modest. To avoid multiple statistical comparisons, a principal component analysis was performed on the two measures of the OCDI, the communication scale of the VABS, and the expressive and receptive language scales of the MSEL. This gave a single factor that accounted for 57% of variance. The mean factor score was 0.14 ± 0.93 for the 60 children in the TD group, and -0.62 ± 1.03 for the 15 children in the SLI group (t_(73) _= 2.79, *P *= 0.007). Thus language status at time 1 is predictive of outcome, but it appears that the effect is variable across measures (Table 3): the MSEL receptive language scale had the strongest association with outcome, followed closely by the VABS communication scale, which is based on parental report of both expressive and receptive language.

**Table 3:** Scores on measures at 18 to 20 months in relation to outcome at 4 years

Measures at 18 to 20 months	Status at 4 years old, mean ± SD		Cohen *d *^b^
	TD^a^, n = 60	SLI, n = 15	
OCDI *z*-scores			
Production	-0.40 ± 0.73	-0.84 ± 1.06	0.56
Comprehension	-0.12 ± 1.18	-0.45 ± 0.84	0.30
VABS, scaled scores^c^			
Communication	101.13 ± 9.7	94.27 ± 9.32	0.72
Daily living	103.07 ± 8.3	98.53 ± 11.24	0.51
Social	95.28 ± 4.96	94.07 ± 4.04	0.26
Motor	98.88 ± 8.35	97.67 ± 4.24	0.16
MSEL *t*-scores^d^			
Expressive language	48.08 ± 11.16	41.13 ± 8.52	0.66
Receptive language	59.42 ± 10.31	50.00 ± 14.35	0.85
Visual reception	56.07 ± 10.65	48.93 ± 10.12	0.69
Motor	49.22 ± 8.02	45.6 ± 9.16	0.44

^a^Abbreviations; MSEL, Mullen Scales of Early Learning; OCDI, Oxford Communicative Development Inventory; SLI, specific language impairment; TD, typical development; VABS, Vineland Adaptive Behavior Scale.

^b^For individual two-tailed *t*-test, uncorrected for multiple comparisons, effect size of 0.57 was significant at *P *= 0.05 level and effect size of 0.83 was significant at *P *= 0.005.

^c^Age scaled score based on mean ± SD of 100 ± 15.

^d^The *t*-score is based on mean ± SD of 50 ± 10.

### Parental characteristics in relation to outcome

Parental characteristics were assessed in relation to child outcome at 4 years (Table 4). Years of maternal education was not related to outcome, but parental score on non-word repetition at time 1 (child 18 to 20 months old) significantly differentiated TD and SLI outcome groups, with large effect sizes. The Pearson correlation between parental non-word repetition at times 1 and 2 was 0.51 (*P *< 0.001), yet only the time 1 result related significantly to the child's outcome. There was a substantial increase in mean score on this test from time 1 to time 2 (t_(70) _= 7.35, *P *< 0.001), but scores at both sessions were below the ceiling value, with around 2% of participants obtaining a maximum score of 46 syllables correct. These findings suggest that the procedural differences in test presentation on the two occasions, and/or prior exposure to the task influenced performance.

**Table 4:** Parental characteristics in relation to the child's outcome at 4 years

**Characteristic^a^**	**Status, mean ± SD**		**t** **(d.f. = 72)**	***P***	**Cohen *d***
				
	**TD, n = 60**	**SLI, n = 14^b^**			
Age of mother at end of FTE, years	22.06 ± 3.6	20.93 ± 2.92	1.09	0.279	0.33
Parent non-word repetition, time 1^c^	11.17 ± 2.63	9.00 ± 2.57	2.79	0.007	0.84
Parent non-word repetition time 2^c^	12.71 ± 1.93	12.53 ± 2.26	0.31	0.758	0.09
Mill-Hill vocabulary, time 2, raw	44.73 ± 7.32	43.07 ± 8.05	0.75	0.457	0.23

^a^Abbreviations: FTE, full-time education; SLI, specific language impairment; TD, typical development.

^b^Parental data not available for one SLI case.

^c ^Scaled score, mean ± 10 ± 3.

### Early predictors of SLI

The question posed at the outset was whether information available about a late talker could be used to predict whether or not the child would go on to have persistent difficulties. To address this question, information from the child and parent measures was combined in a stepwise discriminant function analysis, which identifies the combination of measures that most effectively discriminates between two groups. The method first selects the variable that best discriminates the two groups, and then identifies further variables that significantly improve prediction. In this regard, it resembles the more familiar stepwise multiple regression, whereby inclusion of a variable in the final function indicates that it contributes significant independent predictive power. The child predictors included in the analysis were the three language measures that were most strongly associated with outcome, that is, the VABS communication score and the two MSEL language scores (Table 3). We decided to enter individual test scores rather than an overall principal component, because existing literature suggested that receptive and expressive measures might have different predictive power. Furthermore, this approach allowed us to see whether a full test battery was needed for optimal prediction, or whether a small subset of tests might be useful. As well as child measures, the parental measure of non-word repetition (time 1), and the binary measure of a family history of language-literacy problems were included in the analysis as predictors. A significant discriminant function was obtained using the variables of parental non-word repetition and MSEL receptive *T-*score (Wilks λ = 0.821, χ^2 ^= 13.8, d.f. = 2, *P *= 0.001). Performance of the discriminant function can be evaluated in terms of specificity and sensitivity, but note that these are in a reciprocal relationship, so that as one increases, the other will decrease. High specificity (that is, accuracy in identifying unaffected cases) was obtained using a cutoff of -1.5 on the discriminant function. This correctly identified 59 of 60 unaffected cases, but sensitivity (that is, accuracy in identifying affected cases) was less good, with only 7 of 15 SLI cases correctly identified, equivalent to a sensitivity of 0.46. The area under the curve (AUC) was 0.79 ± 0.062. Crossvalidation was performed using a 'leave-one-out' analysis, in which each case is classified on the basis of a function derived from all the other cases, and this gave identical results.

Because the number of cases with SLI outcome was small, and data on parental non-word repetition was missing for one case, we ran the discriminant analysis again to confirm the findings further, this time omitting the non-word repetition variable. The analysis this time selected the MSEL receptive t-score and the family history variable to give a single discriminant function (Wilks λ = 0.832, χ^2 ^= 13.2, d.f. = 2, *P *= 0.001). With a cutoff of -0.82 on the discriminant function, 10 of 15 SLI cases and 53 of 60 typical language cases were correctly predicted, corresponding to a specificity of 0.88 and sensitivity of 0.67. The AUC was 0.76 ± 0.081. Once again, the crossvalidation 'leave-one-out' analysis gave the same results. Scrutiny of the data indicated that for children with no family history, an MSEL receptive *t*-score that was 1 SD below average (≤40) was predictive of SLI, whereas for those with family history, a good outcome was predicted only when the MSEL receptive t-score was above average (≥61).

In the final analysis, we scrutinized individual cases for which prediction was inaccurate, to determine whether they had any distinctive characteristics when first seen. The first discriminant function, including parental non-word repetition, was slightly more accurate than the second, and so was used for this purpose. Using the optimal cutoff on the function, only one case with typical development was misdiagnosed by the function. This child had scaled scores on the MSEL receptive and expressive language tests that were 2 SD below the mean, whereas the score for visual reception was above average. Scrutiny of her notes indicated that the child was a late talker who had a broken arm when seen at 20 months, with consequent restriction of movement. She was also reported to be very shy. It seems possible that the validity of the initial assessment may have been compromised by these factors.

There were also eight children with SLI who were not identified by the discriminant function. The question arose as to whether these might be marginal cases who narrowly missed detection, but this was not the case. The mean discriminant score for these 'unpredicted' SLI cases was -0.08 ± 0.48 compared with 0.23 ± 0.93 for those with a correct prediction of typical development, t (65) = 0.92, p = .36. Further scrutiny of the 20-month-old test data revealed no indication of deficits in these children at that age. This raises the issue of whether these may be children in whom SLI was misdiagnosed at 4 years of age. This seemed unlikely, as four of these eight children had parental concern and/or speech and language therapy at 4 years, compared with three of seven of those for whom SLI was predicted. To look at this issue in more detail, and see whether the constellation of language scores differentiated between the groups, a multivariate analysis of variance was conducted, contrasting SLI children subdivided according to whether their SLI status had been correctly predicted by the discriminant function, using the variables at 4 years of age (Table 1; for data for individual cases, see Table 5). Missing values were estimated using the mean for two data points. There were no significant differences between the groups, exact *F*_(9,5) _= 0.99, *P *= 0.533). Finally, we considered whether there might be environmental factors that distinguished between those SLI cases that were predictable by the discriminant function and those that were not. In particular, it seemed possible that social disadvantage might exert an effect between 2 and 4 years of age, leading to SLI in some cases. However, there was no evidence for this: the 'predicted' and 'unpredicted' cases of SLI were similar in maternal education and family size. On the measure of parental vocabulary, the Mill-Hill, there was in fact a trend for higher *s*cores in those with 'unpredicted' SLI, (mean 46.8 ± 3.86), than for 'predicted' cases, (mean 39.3 ± 8.16), although this was not significant (t(_12) _= 1.94, *P *= 0.077).

**Table 5:** Individual test scores for children who had specific language impairment at 4 years of age

**ID^a ^no**.	63	158	238	122	143	252	132	355	268	69	195	180	155	323	349
Predicted outcome	SLI	SLI	SLI	SLI	SLI	SLI	SLI	TD	TD	TD	TD	TD	TD	TD	TD
Discriminant score	-2.86	-2.49	-1.99	-1.79	-1.68	-1.64	-1.58	-0.84	-0.73	-0.11	-0.02	0.05	0.20	0.23	0.59
Sex	Boy	Boy	Boy	Boy	Girl	Boy	Boy	Girl	Boy	Boy	Boy	Girl	Girl	Girl	Boy
Positive family history	1	1	1	1	0	0	1	1	0	0	0	1	0	1	0
Parent non-word repetition T1 ss	5	5	10	5	9	12	-	8	12	10	9	10	9	9	13
Block design scaled	9	17	10	10	12	7	12	10	10	12	12	8	15	11	12
Matrices scaled	8	8	8	12	8	10	7	8	10	13	11	8	12	7	7
Language battery^b^															
BAS comprehension	0.92	-0.81	0.10	0.61	-**1.17**	-**2.05**	-0.81	-0.61	0.10	0.10	-**1.17**	0.10	0.92	0.10	-0.81
BAS naming	1.41	0.20	-0.41	0.61	-0.41	-0.99	**-2.33**	0.99	-0.10	1.41	-0.41	0.61	1.41	0.61	1.41
GAPS sentence repetition	0.88	-0.84	-0.47	0.00	0.00	-**1.48**	-0.47	-0.36	0.28	-0.36	-**1.08**	2.00	0.28	-**1.08**	0.28
GAPS non-word repetition	0.44	-**1.41**	-	-0.44	-**1.75**	-**1.75**	-0.95	-**1.41**	2.00	-**1.41**	-**1.75**	-0.44	-0.44	-0.95	2.00
Bus story information	-**1.53**	-0.67	-**1.20**	-**1.07**	-**2.20**	-**1.93**	-**2.07**	-0.07	-**2.07**	-0.20	-**1.33**	-**1.67**	-**1.93**	-**2.20**	-**2.07**
Bus story sentence length	-**1.27**	-0.87	-0.40	-0.87	-0.40	-**1.27**	-**1.27**	0.60	-0.87	1.00	-0.87	0.60	-**1.27**	-**1.80**	-**1.27**
CCC-2 GCC	-0.13	-**1.04**	-0.03	-**1.28**	-0.58	**-1.08**	-0.13	-0.95	**-1.64**	-**1.34**	-**1.64**	0.25	-**1.04**	-	-**1.04**
TEGI 3rd singular^b^	1.00	**0.11**	**0.00**	**0.43**	0.90	**0.00**	0.63	0.78	1.00	**0.50**	**0.44**	**0.67**	1.00	1.00	0.86
TEGI past tense	**0.67**	0.88	0.86	0.94	0.87	**0.29**	0.72	**0.13**	0.75	0.89	**0.18**	0.94	0.88	0.87	1.00

^a^Abbreviations: BAS, British Ability Scales; CCC-2, Children's Communication Checklist, version 2; GAPS, Grammar and Phonology Screening Test; GCC, general communication composite; ID, identification; SLI, specific language impairment; TD, typical development; TEGI, Test of Early Grammatical Impairment.

^b^All data are *z*-scores, except for TEGI, where scores are proportions.

The conclusions from this study are remarkably similar to those obtained by Reilly *et al*. [23] with a much larger epidemiological sample. That group also found that a family history of speech and language problems predicted SLI, and that adding the child's language status at 2 years significantly improved the prediction. In their sample, maternal vocabulary and education also predicted the child's outcome at 4 years. The differences between our results and theirs may reflect the fact that theirs was a population sample that included a broad range of social backgrounds, whereas ours was a convenience sample selected predominantly from parents who had signed up as research volunteers, and was biased to well-educated middle-class parents.

It has previously been found that if reliance is placed solely on very early child-language measures, both sensitivity and specificity of prediction of outcome are poor, with many late talkers catching up with their peers [22,45]. However, when familial information is combined with information on early language difficulties, prediction is improved [23,24]. In the present study, we found that by including direct measurement of non-word repetition in a parent, we obtained good specificity, but sensitivity remained low for predicting SLI at 4 years of age. In other words, when parental non-word repetition was included as a predictor, poor prediction arose because many children who had been predicted to have typical development went on to develop SLI. The pattern was less striking when family history was used as a predictor; in this case both sensitivity and specificity of prediction dropped, but here too, at the optimal cutoff on the discriminant function, typical development was predicted for some children who subsequently developed SLI. Caution must be adopted in interpreting the sensitivity and specificity values obtained with this small sample, as recategorization of one or two cases could lead to substantial changes in proportions. Nevertheless, it is noteworthy that similar findings have been reported by others [17,21,22]. Dale *et al*. [17] suggested that 'unpredicted' cases of SLI might be an artefact of the methods used, which involved creating binary categories from a continuous distribution. Nevertheless, in our sample, these 'unpredicted' children with SLI were not close to the boundaries for impairment, either in terms of the discriminative function, or in terms of their test scores at 4 years old. Another explanation was proposed by authors of the ELVS study [23], who suggested that environmental factors start to contribute to language impairments as children grow older. There was no evidence of this in our sample: maternal education, family size or parental vocabulary did not distinguish 'predicted' from 'unpredicted' cases of SLI. However, the small sample size and restricted range of social backgrounds needs to be taken into account when evaluating this result.

An anomalous aspect of the current data is the finding that parental non-word repetition was a good predictor of the child's outcome when it was measured on the first testing occasion, when the child was 20 months old, but not on the second occasion, when the child was 4 years old. As noted above, there was a procedural difference between the two occasions, associated with a significant improvement in scores at the second test session. The change in procedure had been instituted after pilot testing revealed concerns about potential distractions when administering the test in the family home. However, the fact that the test no longer predicted the child's outcome when presented this way suggests that attention, as well as phonological perception and memory, may be a key factor determining non-word repetition deficits in familial SLI. The traditional method of administration requires the participant to attend to a recorded series of non-words, which are interspersed with 5-second periods of silence. Problems in maintaining attention are the likely explanation of poor performance by some parents tested in this way. Further research is needed to determine how far procedural changes in test administration affect the performance of those with SLI and their parents. Practice effects are also possible: it may be that after the first testing session, differences between groups are ironed out because there is learning of the test items. However, this seems less likely given the long interval between test sessions and the fact that no feedback about accuracy was provided.

The results from this study have clinical implications, although interpretation must be cautious, given the fact that the sample was small and included few cases of low SES. Nevertheless, the findings suggest that where there is a family history of language/literacy problems in a first-degree relative, it is appropriate to monitor the child carefully unless there is clear evidence of above-average language development. For children with no such family history, a receptive language score of 1 SD or more below age level at 20 months is predictive of persistent language difficulties. At first glance, our findings seem discrepant with those of Westerlund *et al*. [21], who found expression at 18 months to be a better predictor of outcome than receptive language. However, that group used parental report of comprehension, which is known to be unreliable. Our result is consistent with the view that low comprehension in a toddler should be a 'red flag' suggestive of poor outcome [19], provided that comprehension is measured by formal assessment of the child. A focus solely on the age at which the child develops a sizeable vocabulary seems misplaced, given that this on its own is a poor predictor of outcome, with many children doing well after a slow start. We suggest it is important to take into account familial factors and child behaviors to ensure that early intervention is most effectively targeted.

## Conclusions

Overall, our results join others in the literature in suggesting that 18 to 20 months of age may be too early for effective screening for language problems because individual children can show rapid acceleration of language skills around this age. Furthermore, some children develop language difficulties by 4 years of age despite normal early vocabulary development. Follow-up studies of older toddlers may help us uncover the optimal age at which to identify children at risk for language difficulties, while minimizing false positives.

## List of abbreviations used

BAS: British Ability Scales; CCC-2: Children's Communication Checklist, version 2; ELVS: Early Language in Victoria Study; FH+: positive family history; GAPS: Grammar and Phonology Screening Test; GCC: General Communication Composite; LNV: low nonverbal; NLI, nonspecific language impairment; OCDI: Oxford Communicative Development Inventory; PIQ: Performance Intelligence Quotient; SALT: speech and language therapy; SLI: specific language impairment; TD: typical development; TEGI: Test of Early Grammatical Impairment; VABS: Vineland Adaptive Behavior Scales; WPPSI: Wechsler Preschool and Primary Scale of Intelligence.

## Competing interests

The authors declare that they have no competing interests.

## Authors' contributions

SM, DM and EL carried out recruitment and assessment of children for the initial assessment, and HW and GH assessed children at 4 years of age. DVMB conceived of and designed the study, carried out data analysis, and wrote the first draft of the manuscript. All authors reviewed relevant literature, discussed interpretation of results, and read and approved the final manuscript.
