# Erratum: Parental phonological memory contributes to prediction of outcome of late talkers from 20 months to 4 years: a longitudinal study of precursors of specific language impairment

**DOI:** 10.1186/s11689-015-9110-0

**Published:** 2015-05-30

**Authors:** Dorothy VM Bishop, Georgina Holt, Elizabeth Line, David McDonald, Sarah McDonald, Helen Watt

**Affiliations:** Department of Experimental Psychology, University of Oxford, South Parks Road, Oxford, OX1 3UD UK

## Erratum

We have discovered an error in our article on “Parental phonological memory contributes to prediction of outcome of late talkers from 20 months to 4 years: a longitudinal study of precursors of specific language impairment” [1]. The account of how OCDI scores were converted to standardized scores (Page 4, column 2, section headed ‘Assessments at 18 to 20 months of age’) is incorrect. Although we explored the use of regression methods to derive norms, we decided against this approach because of skew in the data. In the final analysis we took existing normative data [2] for the age range 18.5–21 months and used the cumulative frequency distribution of word production scores to identify a cutoff. We used OCDI items 13–416 (i.e. excluding animal sounds), as many children in the normative sample did not have data on items 1–12. The cumulative frequency for 11 words was 16.4 % and for 10 words was 12.4 %, and so we used a cutoff of 10 words or less to correspond to one SD below average.

The analyses in the article were computed using this cutoff, and so none of the results or conclusions are altered: the correction is made to ensure that the description of the method in the article matches what was done.
